# Supplementary material for: Targeted transcriptome analysis using synthetic long read sequencing uncovers isoform reprograming in the progression of colon cancer
Source: Commun Biol. 2021 Apr 27;4:506. doi: 10.1038/s42003-021-02024-1 (PMC8079361; doi:10.1038/s42003-021-02024-1)
Supplement: Supplementary file 2 — Supplementary Information [file 42003_2021_2024_MOESM2_ESM.pdf]

# Targeted Transcriptome Analysis using Synthetic Long Read Sequencing Uncovers Isoform Reprograming in the Progression of Colon Cancer

Silvia Liu[*, 1,2,3], Indira Wu[*,4], Yan-Ping Yu[1,2,3], Michael Balamotis[4], Baoguo Ren[1,2], Tuval Ben Yehezkel[+,4] and Jian-Hua Luo[+,1,2,3]

[1]Department of Pathology, University of Pittsburgh School of Medicine, 3550 Terrace Street, Pittsburgh, PA 15261, USA

[2]High Throughput Genome Center, University of Pittsburgh School of Medicine, 3550 Terrace Street, Pittsburgh, PA 15261, USA

[3]Pittsburgh Liver Research Center, University of Pittsburgh School of Medicine, 3550 Terrace Street, Pittsburgh, PA 15261, USA

[4]Loop Genomics, Inc., 5941 Optical Court, San Jose, CA 95138, USA

*- Contributed equally

+ - Co-corresponding authors: Jian-Hua Luo, 3550 Terrace Street, Scaife S-728, Department of Pathology, University of Pittsburgh School of Medicine, Pittsburgh, PA 15261; E-mail: luoj@upmc.edu; Tuval Ben Yehezkel, 5941 Optical Court, San Jose, CA 95138; E-mail: tuval@loopgenomics.com.

**Supplementary Figure S1. SLR Short-Read Coverage Uniformity.** Illumina short read coverage is plotted along the length of de novo reconstructed SLRs. SLR lengths were normalized to 100 bins (x axis) and the average number of short reads per bin is plotted (y axis).

**Supplementary Figure S2. Isoform expression of CD44.** Normalized CD44 isoform expression. Arrows indicate isoforms that are overexpressed in cancers and metastases.

**Supplementary Figure S3. Isoform expression of ATP1A1.** Normalized ATP1A1 isoform expression. Arrows indicate isoforms that are overexpressed in cancers.

**Supplementary Figure S4. Taqman qRT-PCR to quantify isoforms of ATP1A1.** Five sets of primers and probes for the indicated isoforms were designed. Taqman qRT-PCRs were performed triplicate to quantify the isoform expression. The results were normalized to the mRNA of β-actin. Three independent experiments were performed for each sample per isoform. Standard deviation is indicated.

**Supplementary Figure S5. Non-switching SNV isoforms do not segregate primary cancer samples and metastasis samples.** Hierarchical clustering between primary colon cancers and metastatic colon cancers based on the quantities of total (top) or non-switching (bottom) non-synonymous single nucleotide variants of all isoforms in each sample. The color reflects SNV rate by fraction. (B) Principal component analyses of primary colon cancers and metastatic colon cancers based on the quantities of total (top) or non-switching (bottom) non-synonymous single nucleotide variants of (A). (C) Pearson's correlation of primary colon cancers and metastatic colon cancers based on the quantities of total (top) or non-switching (bottom) non-

synonymous single nucleotide variants of (A). The color reflects Pearson's correlation coefficient for the pairing samples.

**Supplementary Figure S6. Schematic diagram of fusion gene screening criteria.**

**Supplementary Figure S7. Validation and screening analyses of STAMBPL1-FAS.** Taqman qRT-PCRs were performed on benign colon tissues adjacent cancer, colon cancer and lymph node metastasis samples using the primers and probes described in the methods. The positions of primers and probes are indicated.

**Supplementary Figure S8. Validation and screening analyses of ZNF124-SMYD3.** Taqman qRT-PCRs were performed on benign colon tissues adjacent cancer, colon cancer and lymph node metastasis samples using the primers and probes described in the methods. The positions of primers and probes are indicated.

**Supplementary Figure S9. Validation and screening analyses of PTPRK-ECHDC1.** Taqman qRT-PCRs were performed on benign colon tissues adjacent cancer, colon cancer and lymph node metastasis samples using the primers and probes described in the methods.

**Supplementary Figure S10. Validation and screening analyses of VAPB-GNAS.** Taqman qRT-PCRs were performed on benign colon tissues adjacent cancer, colon cancer and lymph node metastasis samples using the primers and probes described in the methods. Taqman qRT-PCRs on β-actin are the controls. The positions of primers and probes are indicated.

**Supplementary Figure S11. Validation and screening analyses of β-actin controls.** Taqman qRT-PCRs were performed on benign colon tissues adjacent cancer, colon cancer and lymph node metastasis samples using the primers and probes described in the methods.

**Supplementary Note 1:** Scripts for bioinformatics analysis

Below is a list of the parameters used for the public domain programs and the LoopSeq pipeline

## De Novo Assembly

SPADES was run from a python script with the follow parameters:
command = spades.py -k 21,33,55,77,99,127 -t 1 --careful --sc --pe1-1 left.fq --pe1-2 right.fq --pe1-s unpaired.fq -o spades_output --disable-gzip-output

https://github.com/ablab/spades

Bankevich, A., Nurk, S., Antipov, D., Gurevich, A. A., Dvorkin, M., Kulikov, A. S., Lesin, V. M., Nikolenko, S. I., Pham, S., Prjibelski, A. D., Pyshkin, A. V., Sirotkin, A. V., Vyahhi, N., Tesler, G., Alekseyev, M. A., & Pevzner, P. A. (2012). SPAdes: a new genome assembly algorithm and its applications to single-cell sequencing. Journal of computational biology : a journal of computational molecular cell biology, 19(5), 455–477. https://doi.org/10.1089/cmb.2012.0021

## Long-read transcriptome analysis

SQANTI was run from the command line with default parameters:

- Copy and paste *contig_list_trimmed.fa for each sample folder
- isoformsFasta holds the prepared reference files such as *.fa and *.gtf from pipeline output
- refGenome = "$REFERENCE_DIR/Homo_sapiens.GRCh37.75.dna.primary_assembly.fa"
- refGTF = "$REFERENCE_DIR/Homo_sapiens.GRCh37.75.gtf"
- gmapIndex = "$REFERENCE_DIR/Homo_sapiens.GRCh37.75.dna.primary_assembly"
- command = sqanti_qc.py $isoformsFasta $refGTF $refGenome -n -x $gmapIndex -t 8

## Short read trimming

Trimmomatics was run from a python script with the follow parameters:
command = ['java -jar ' + pipeline.prog_path + '/Trimmomatic-0.36/trimmomatic-0.36.jar PE -threads 32 -trimlog ' + trim_log_file + ' ','../' + pipeline.input_params['raw_file_R1'], '../' + pipeline.input_params['raw_file_R2'],' '.join(trim_output_files), 'ILLUMINACLIP:' + pipeline.prog_path + '/JAStrim.fa:2:40:14:3:true TRAILING:20 SLIDINGWINDOW:4:15 MINLEN:36']

## Alignment of long reads to reference by BLAST

BLAST was run from a python script with default parameters:
blastn -db <reference database> -query contig_list.fa -perc_identity=<pct_id_threshold> -qcov_hsp_perc=<qcov_threshold> -max_target_seqs=<max_seqs> -num_threads=16 -outfmt=6 > mapping.blst

## q score model

```
## ad: allelic depth (coverage) of a given position
## ref: reference allele in consensus sequence
## alt: alternative allele(s)
ad_ref = ad_mat[...,0]
ad_alt = ad_mat[...,1]
ad_totals = ad_mat.sum(axis=1)
qscore = np.array([10  for i in range(ad_mat.shape[0])])

## Positions covered by 1 read, scale phred score of read into [0,30] range.
## rawqual is Illumina phred score for the single nucleotide in the single read covering this position
idx = np.logical_and(ad_ref == 1, ad_alt == 0); qscore[idx] = np.round((rawqual[idx]) * (30.0/40.0))

## Positions covered by >1 read with no alternative allele
idx = np.logical_and(ad_ref == 2, ad_alt == 0); qscore[idx] = 30
idx = np.logical_and(ad_ref >= 3, ad_alt == 0); qscore[idx] = 30+ad_ref[idx]
idx = np.logical_and(ad_ref > 10, ad_alt == 0); qscore[idx] = 41

## Positions covered by >10 reads with alternative allele(s)
idx = np.logical_and(ad_ref > 10, ad_alt == 1); qscore[idx] = -40 * np.log10(ad_alt[idx] / ad_totals[idx])
idx = np.logical_and(ad_ref > 10, ad_alt == 2); qscore[idx] = -40 * np.log10(ad_alt[idx] / ad_totals[idx])
idx = np.logical_and(ad_ref > 10, ad_alt > 2); qscore[idx] = -40 * np.log10(ad_alt[idx] / ad_totals[idx])

## Positions covered by <=10 reads with alternative allele(s)
idx = np.logical_and(ad_ref <= 10, ad_alt > 0); qscore[idx] = -40 * np.log10(ad_alt[idx] / ad_totals[idx])
idx = np.logical_and(ad_ref <= 5, ad_alt > 0); qscore[idx] = -40 * np.log10(ad_alt[idx] / ad_totals[idx])
idx = np.logical_and(ad_ref <= 3, ad_alt > 0); qscore[idx] = -40 * np.log10(ad_alt[idx] / ad_totals[idx])

## Transform probability of homozygosity to phred score range [10,41]
## qual is bcftools estimate for probability of homozygosity at a given position
qual_qscore = -10 * np.log10(qual) + 10

# Take maximum score across probability model and arbitrary
# assignment.  Trim quality scores that are above 41.
qscore = np.maximum(qual_qscore,qscore)
qscore = np.minimum(41, qscore)
```

## Alignment of long read to reference by STARlong

STARlong --runMode alignReads --runThreadN 2 --genomeDir $genomeDir --readFilesIn $InFile --outFileNamePrefix $outPath/$sample".STAR." --outSAMtype BAM SortedByCoordinate --quantMode GeneCounts --outSAMattributes NH HI NM MD --readNameSeparator space --outFilterMultimapScoreRange 1 --outFilterMismatchNmax 2000 --scoreGapNoncan -20 --scoreGapGCAG -4 --scoreGapATAC -8 --scoreDelOpen -1 --scoreDelBase -1  --scoreInsOpen -1 --scoreInsBase -1  --alignEndsType Local --seedSearchStartLmax 50 --seedPerReadNmax 100000 --seedPerWindowNmax 1000 --alignTranscriptsPerReadNmax 100000 --alignTranscriptsPerWindowNmax 10000

Parameter setting reference:
Križanović, Krešimir, et al. "Evaluation of tools for long read RNA-seq splice-aware alignment." Bioinformatics 34.5 (2018): 748-754.

https://academic.oup.com/bioinformatics/article/34/5/748/4562330

**Alignment of long read to reference by Minimap2**

```
## minimap2 for alignment, default parameter setting
minimap2 -ax splice $refFile $InFile | samtools view -Sb | samtools sort -o $BAMfile
samtools index $BAMfile
```

**SNV calling**

```
## mpileup for SNV calling, based on Minimap2 alignment files
bcftools mpileup -d 1000 -f $refFile $BAMfile > $outPathM/mpileup.txt

## file formating
cat $outPathM/mpileup.txt | sed '/^#/d' |  awk -F "\t" ' $5 ~ /,/ {print}' > $outPathM/mpile
up_var.txt
cat $outPathM/mpileup_var.txt | sed '/^#/d' |  awk -F "\t" '{print $1 "\t" $2 "\t" $4 }' > $
outPathM/col123.txt
cat $outPathM/mpileup_var.txt | sed '/^#/d' |  awk -F "\t" '{print $5}' | sed 's/,<\*>//g' >
 $outPathM/col4.txt
cat $outPathM/mpileup_var.txt | sed '/^#/d' |  awk -F "\t" '{print $8}' | awk -F ";" '{print
 $2}' | sed 's/I16=//g' | awk -F "," '{print expr ($1 + $2) "\t" expr ($3 + $4)}' > $outPath
M/col56.txt
paste $outPathM/col123.txt $outPathM/col4.txt $outPathM/col56.txt > $outPathM/call_var_all.t
xt
```

**Gene and isoform quantification based on SQANTI output and LoopSeq stat files**
See getCount.r script for details

```
#### getCount.r ####
###################### TP08-S00678LY, 1L

rm(list=ls())

res=read.csv("../data/1064_JianHua_1_pipeline_output/sample_TP08-
S00678LY/stats_trimmed.csv",header=T,as.is=T)
dim(res) # 204081     15

## remove molecules without gene annotation
NAind=which(res$gene_name_human_rna=="")
```

```
res=res[-NAind,]
dim(res) # 200468     15

rownames(res)=res$molecule_id

## barcode stat file
stat=read.csv("../data/stat/1064_stats.csv",header=T,as.is=T)
keepInd=which(stat$molecule_id!="")

df=data.frame(molecule_id=stat$molecule_id[keepInd],
read_count=stat$read_count[keepInd],
            gene=res[stat$molecule_id[keepInd],"gene_name_human_rna"],
            isoform=res[stat$molecule_id[keepInd],"ref_id_human_rna"],
            stringsAsFactors = F)
df[is.na(df$isoform),"isoform"]="NA"  # correct NA isoform
df=cbind(df,isoName=paste(df$gene,df$isoform,sep="__"))
dim(df)  # 204081      5

## gene count
dfSplitGene=split(df,df$gene)
geneCount=sapply(dfSplitGene,function(x) return(sum(x[,2])))
length(geneCount)  # 3808

## isoform count
dfSplitIso=split(df,df$isoName)
isoCount=sapply(dfSplitIso,function(x) return(sum(x[,2])))
length(isoCount)  # 7061  including NA

gene=sapply(strsplit(names(isoCount),split="__"), function(x) return(x[1]))

tab=data.frame(Gene=gene,GeneCount=as.numeric(geneCount[gene]),
             Isoform=sapply(strsplit(names(isoCount),split="__"), function(x) return(x[2])),
             IsoformCount=as.numeric(isoCount))

write.csv(tab,file="count/sample_TP08-S00678LY.count.csv",row.names=F,quote=F)

## plot
hist(log(geneCount,base=10),breaks=50, xlab="log10 (Num of long-reads per gene)",
ylab="Gene count",main=NA)

hist(log(isoCount,base=10),breaks=50, xlab="log10 (Num of long-reads per isoform)",
ylab="Isoform count",main=NA)
```

```
##################### TP08-S00678T, 1T

rm(list=ls())

res1=read.csv("../data/1065_JianHua_2_pipeline_output/sample_TP08-S00678T/stats_trimmed.csv",header=T,as.is=T)
dim(res1) # 210604     15
res1$molecule_id=gsub("SAMPLE_1_MOLECULE","SAMPLE_1065_MOLECULE",res1$molecule_id)

res2=read.csv("../data/1072_JianHua_2_75C_pipeline_output/sample_TP08-S00678T/stats_trimmed.csv",header=T,as.is=T)
dim(res2) # 176889     15
res2$molecule_id=gsub("SAMPLE_1_MOLECULE","SAMPLE_1072_MOLECULE",res2$molecule_id)

res3=read.csv("../data/1073_JianHua_2_78C_pipeline_output/sample_TP08-S00678T/stats_trimmed.csv",header=T,as.is=T)
dim(res3) # 25423    15
res3$molecule_id=gsub("SAMPLE_1_MOLECULE","SAMPLE_1073_MOLECULE",res3$molecule_id)

res=rbind(res1,res2,res3)
dim(res)  # 412916     15
rownames(res)=res$molecule_id

## remove molecules without gene annotation
NAind=which(res$gene_name_human_rna=="")
res=res[-NAind,]
dim(res) # 409115     15

## barcode stat file
stat1=read.csv("../data/stat/1065_stats.csv",header=T,as.is=T)
keepInd=which(stat1$molecule_id!="")
stat1=stat1[keepInd,]
stat1$molecule_id=gsub("SAMPLE_1_MOLECULE","SAMPLE_1065_MOLECULE",stat1$molecule_id)

stat2=read.csv("../data/stat/1072_stats.csv",header=T,as.is=T)
keepInd=which(stat2$molecule_id!="")
stat2=stat2[keepInd,]
stat2$molecule_id=gsub("SAMPLE_1_MOLECULE","SAMPLE_1072_MOLECULE",stat2$molecule_id)
```

```
stat3=read.csv("../data/stat/1073_stats.csv",header=T,as.is=T)
keepInd=which(stat3$molecule_id!="")
stat3=stat3[keepInd,]
stat3$molecule_id=gsub("SAMPLE_1_MOLECULE","SAMPLE_1073_MOLECULE",stat3$molecule
_id)

stat=rbind(stat1,stat2,stat3)

sum(res$molecule_id%in%stat$molecule_id)  # 409115

df=data.frame(molecule_id=stat$molecule_id, read_count=stat$read_count,
            gene=res[stat$molecule_id,"gene_name_human_rna"],
            isoform=res[stat$molecule_id,"ref_id_human_rna"],
            stringsAsFactors = F)
df[is.na(df$isoform),"isoform"]="NA"  # correct NA isoform
df=cbind(df,isoName=paste(df$gene,df$isoform,sep="__"))
dim(df)  # 412916      5

## gene count
dfSplitGene=split(df,df$gene)
geneCount=sapply(dfSplitGene,function(x) return(sum(x[,2])))
length(geneCount)  # 5139

## isoform count
dfSplitIso=split(df,df$isoName)
isoCount=sapply(dfSplitIso,function(x) return(sum(x[,2])))
length(isoCount)  # 5962  including NA

gene=sapply(strsplit(names(isoCount),split="__"), function(x) return(x[1]))

tab=data.frame(Gene=gene,GeneCount=as.numeric(geneCount[gene]),
             Isoform=sapply(strsplit(names(isoCount),split="__"), function(x) return(x[2])),
             IsoformCount=as.numeric(isoCount))

write.csv(tab,file="count/sample_TP08-S00678T.count.csv",row.names=F,quote=F)




###################### TP08-S00678N, 1N

rm(list=ls())
```

```
res1=read.csv("../data/result3/1854_1183_mRNA_JianHua_AB107/1854_output/1854_sample
_TP08-S00678T-NORMAL_a/1854_sample_TP08-S00678T-
NORMAL_a_stats_trimmed.csv",header=T,as.is=T)
dim(res1) # 123281     14

res2=read.csv("../data/result3/1854_1183_mRNA_JianHua_AB107/1854_output/1854_sample
_TP08-S00678T-NORMAL_b/1854_sample_TP08-S00678T-
NORMAL_b_stats_trimmed.csv",header=T,as.is=T)
dim(res2)  # 133766     14

res=rbind(res1,res2)
dim(res)  # 257047     14
rownames(res)=res$molecule_id

## remove molecules without gene annotation
NAind=which(res$gene_name_human_rna=="")
res=res[-NAind,]
dim(res) # 252474     14

## barcode stat file
stat1=read.csv("../data/shortRead3/1854/1854_sample_TP08-S00678T-
NORMAL_a_stats.csv",header=T,as.is=T)
keepInd=which(stat1$molecule_id!="")
stat1=stat1[keepInd,]

stat2=read.csv("../data/shortRead3/1854/1854_sample_TP08-S00678T-
NORMAL_b_stats.csv",header=T,as.is=T)
keepInd=which(stat2$molecule_id!="")
stat2=stat2[keepInd,]

stat=rbind(stat1,stat2)

sum(res$molecule_id%in%stat$molecule_id)  # 252474

df=data.frame(molecule_id=stat$molecule_id, read_count=stat$read_count,
          gene=res[stat$molecule_id,"gene_name_human_rna"],
          isoform=res[stat$molecule_id,"ref_id_human_rna"],
          stringsAsFactors = F)
df[is.na(df$isoform),"isoform"]="NA"  # correct NA isoform
df=cbind(df,isoName=paste(df$gene,df$isoform,sep="__"))
dim(df)  # 257047      5

## gene count
dfSplitGene=split(df,df$gene)
```

```
geneCount=sapply(dfSplitGene,function(x) return(sum(x[,2])))
length(geneCount)  # 4886

## isoform count
dfSplitIso=split(df,df$isoName)
isoCount=sapply(dfSplitIso,function(x) return(sum(x[,2])))
length(isoCount)  # 8882  including NA

gene=sapply(strsplit(names(isoCount),split="__"), function(x) return(x[1]))

tab=data.frame(Gene=gene,GeneCount=as.numeric(geneCount[gene]),
            Isoform=sapply(strsplit(names(isoCount),split="__"), function(x) return(x[2])),
            IsoformCount=as.numeric(isoCount))

write.csv(tab,file="count/sample_TP08-S00678N.count.csv",row.names=F,quote=F)






###################### TP09-P199T, 2T

rm(list=ls())

res=read.csv("../data/1066_JianHua_3_pipeline_output/sample_TP09-
P199T/stats_trimmed.csv",header=T,as.is=T)
dim(res) # 243296     15

## remove molecules without gene annotation
NAind=which(res$gene_name_human_rna=="")
res=res[-NAind,]
dim(res) # 240787    15

rownames(res)=res$molecule_id

## barcode stat file
stat=read.csv("../data/stat/1066_stats.csv",header=T,as.is=T)
keepInd=which(stat$molecule_id!="")

df=data.frame(molecule_id=stat$molecule_id[keepInd],
read_count=stat$read_count[keepInd],
           gene=res[stat$molecule_id[keepInd],"gene_name_human_rna"],
```

```
            isoform=res[stat$molecule_id[keepInd],"ref_id_human_rna"],
            stringsAsFactors = F)
df[is.na(df$isoform),"isoform"]="NA"  # correct NA isoform
df=cbind(df,isoName=paste(df$gene,df$isoform,sep="__"))
dim(df)  # 243296      5

## gene count
dfSplitGene=split(df,df$gene)
geneCount=sapply(dfSplitGene,function(x) return(sum(x[,2])))
length(geneCount)  # 5170

## isoform count
dfSplitIso=split(df,df$isoName)
isoCount=sapply(dfSplitIso,function(x) return(sum(x[,2])))
length(isoCount)  # 9389  including NA

gene=sapply(strsplit(names(isoCount),split="__"), function(x) return(x[1]))

tab=data.frame(Gene=gene,GeneCount=as.numeric(geneCount[gene]),
             Isoform=sapply(strsplit(names(isoCount),split="__"), function(x) return(x[2])),
             IsoformCount=as.numeric(isoCount))


write.csv(tab,file="count/sample_TP09-P199T.count.csv",row.names=F,quote=F)


###################### TP10-S0582LY, 2L

rm(list=ls())

res1=read.csv("../data/1067_JianHua_4_pipeline_output/sample_TP10-
S0582LY/stats_trimmed.csv",header=T,as.is=T)
dim(res1) # 229282     15
res1=res1[,!colnames(res1)%in%"cluster_dada2"]
res1$molecule_id=gsub("SAMPLE_1_MOLECULE","SAMPLE_1067_MOLECULE",res1$molecule_i
d)
res1$molecule_id=gsub("SAMPLE_1_MOLECULE","1067_SAMPLE_TP10-
S0582_L_MOLECULE",res1$molecule_id)

res2=read.csv("../data/result3/1855_1184_mRNA_JianHua_AB108/1855_output/1855_sample
_TP10-S0582LY-tumor_a/1855_sample_TP10-S0582LY-tumor_a_stats_trimmed.csv",
header=T,as.is=T)
dim(res2)  # 178104     14
```

```
res3=read.csv("../data/result3/1855_1184_mRNA_JianHua_AB108/1855_output/1855_sample
_TP10-S0582LY-tumor_b/1855_sample_TP10-S0582LY-tumor_b_stats_trimmed.csv",
header=T,as.is=T)
dim(res3)  # 194836     14

res4=read.csv("../data/result3/1856_1185_mRNA_JianHua_AB108/1856_output/1856_sample
_TP10-S0582LY-tumor_c/1856_sample_TP10-S0582LY-tumor_c_stats_trimmed.csv",
header=T,as.is=T)
dim(res4)  # 178502     14

res5=read.csv("../data/result3/1856_1185_mRNA_JianHua_AB108/1856_output/1856_sample
_TP10-S0582LY-tumor_d/1856_sample_TP10-S0582LY-tumor_d_stats_trimmed.csv",
header=T,as.is=T)
dim(res5)  # 195602     14

res=rbind(res1,res2,res3,res4,res5)
dim(res)  # 976326     14
rownames(res)=res$molecule_id

## remove molecules without gene annotation
NAind=which(res$gene_name_human_rna=="")
res=res[-NAind,]
dim(res) # 965223     14

## stat
stat1=read.csv("../data/stat/1067_stats.csv",header=T,as.is=T)
keepInd=which(stat1$molecule_id!="")
stat1=stat1[keepInd,]
stat1=stat1[,!colnames(stat1)%in%"cluster_dada2"]
stat1$molecule_id=gsub("SAMPLE_1_MOLECULE","SAMPLE_1067_MOLECULE",stat1$molecule
_id)

stat2=read.csv("../data/shortRead3/1855/1855_sample_TP10-S0582LY-
tumor_a_stats.csv",header=T,as.is=T)
keepInd=which(stat2$molecule_id!="")
stat2=stat2[keepInd,]

stat3=read.csv("../data/shortRead3/1855/1855_sample_TP10-S0582LY-
tumor_b_stats.csv",header=T,as.is=T)
keepInd=which(stat3$molecule_id!="")
stat3=stat3[keepInd,]

stat4=read.csv("../data/shortRead3/1856/1856_sample_TP10-S0582LY-
tumor_c_stats.csv",header=T,as.is=T)
```

```
keepInd=which(stat4$molecule_id!="")
stat4=stat4[keepInd,]

stat5=read.csv("../data/shortRead3/1856/1856_sample_TP10-S0582LY-
tumor_d_stats.csv",header=T,as.is=T)
keepInd=which(stat5$molecule_id!="")
stat5=stat5[keepInd,]

stat=rbind(stat1,stat2,stat3,stat4,stat5)

sum(res$molecule_id%in%stat$molecule_id)  # 965223

df=data.frame(molecule_id=stat$molecule_id, read_count=stat$read_count,
            gene=res[stat$molecule_id,"gene_name_human_rna"],
            isoform=res[stat$molecule_id,"ref_id_human_rna"],
            stringsAsFactors = F)
df[is.na(df$isoform),"isoform"]="NA"  # correct NA isoform
df=cbind(df,isoName=paste(df$gene,df$isoform,sep="__"))
dim(df)  # 976326      5

## gene count
dfSplitGene=split(df,df$gene)
geneCount=sapply(dfSplitGene,function(x) return(sum(x[,2])))
length(geneCount)  # 6162

## isoform count
dfSplitIso=split(df,df$isoName)
isoCount=sapply(dfSplitIso,function(x) return(sum(x[,2])))
length(isoCount)  # 12720  including NA

gene=sapply(strsplit(names(isoCount),split="__"), function(x) return(x[1]))

tab=data.frame(Gene=gene,GeneCount=as.numeric(geneCount[gene]),
             Isoform=sapply(strsplit(names(isoCount),split="__"), function(x) return(x[2])),
             IsoformCount=as.numeric(isoCount))

write.csv(tab,file="count/sample_TP10-S0582LY.count.csv",row.names=F,quote=F)




###################### TP10-S1000LY, 3L

rm(list=ls())
```

```
res=read.csv("../data/1068_JianHua_5_pipeline_output/sample_TP10-
S1000LY/stats_trimmed.csv",header=T,as.is=T)
dim(res) # 231091     15

## remove molecules without gene annotation
NAind=which(res$gene_name_human_rna=="")
res=res[-NAind,]
dim(res) # 228351     15

rownames(res)=res$molecule_id

## barcode stat file
stat=read.csv("../data/stat/1068_stats.csv",header=T,as.is=T)
keepInd=which(stat$molecule_id!="")

df=data.frame(molecule_id=stat$molecule_id[keepInd],
read_count=stat$read_count[keepInd],
            gene=res[stat$molecule_id[keepInd],"gene_name_human_rna"],
            isoform=res[stat$molecule_id[keepInd],"ref_id_human_rna"],
            stringsAsFactors = F)
df[is.na(df$isoform),"isoform"]="NA"  # correct NA isoform
df=cbind(df,isoName=paste(df$gene,df$isoform,sep="__"))
dim(df)  # 231091     5

## gene count
dfSplitGene=split(df,df$gene)
geneCount=sapply(dfSplitGene,function(x) return(sum(x[,2])))
length(geneCount)  # 4608

## isoform count
dfSplitIso=split(df,df$isoName)
isoCount=sapply(dfSplitIso,function(x) return(sum(x[,2])))
length(isoCount)  # 8630  including NA

gene=sapply(strsplit(names(isoCount),split="__"), function(x) return(x[1]))

tab=data.frame(Gene=gene,GeneCount=as.numeric(geneCount[gene]),
             Isoform=sapply(strsplit(names(isoCount),split="__"), function(x) return(x[2])),
             IsoformCount=as.numeric(isoCount))

write.csv(tab,file="count/sample_TP10-S1000LY.count.csv",row.names=F,quote=F)
```

```
###################### TP10-S1000T, 3T

rm(list=ls())

res1=read.csv("../data/1069_JianHua_6_pipeline_output/sample_TP10-S1000T/stats_trimmed.csv",header=T,as.is=T)
dim(res1) # 241984     15
res1=res1[,!colnames(res1)%in%"cluster_dada2"]
res1$molecule_id=gsub("SAMPLE_1_MOLECULE","SAMPLE_1069_MOLECULE",res1$molecule_id)
#res1$molecule_id=gsub("SAMPLE_1_MOLECULE","1069_SAMPLE_TP10-S1000_T_MOLECULE",res1$molecule_id)

res2=read.csv("../data/result3/1853_1182_mRNA_JianHua_AB106/1853_output/1853_sample_TP10-S1000T-tumor_c/1853_sample_TP10-S1000T-tumor_c_stats_trimmed.csv",header=T,as.is=T)
dim(res2)  # 162606     14

res3=read.csv("../data/result3/1853_1182_mRNA_JianHua_AB106/1853_output/1853_sample_TP10-S1000T-tumor_d/1853_sample_TP10-S1000T-tumor_d_stats_trimmed.csv",header=T,as.is=T)
dim(res3)  # 162606     14

res4=read.csv("../data/result3/1857_1181_mRNA_JianHua_AB106_rerun/1857_output/1857_sample_TP10-S1000T-tumor_a/1857_sample_TP10-S1000T-tumor_a_stats_trimmed.csv",header=T,as.is=T)
dim(res4)  # 162888     14

res5=read.csv("../data/result3/1857_1181_mRNA_JianHua_AB106_rerun/1857_output/1857_sample_TP10-S1000T-tumor_b/1857_sample_TP10-S1000T-tumor_b_stats_trimmed.csv",header=T,as.is=T)
dim(res5)  # 227466     14

res=rbind(res1,res2,res3,res4,res5)
dim(res)  # 1022111     14
rownames(res)=res$molecule_id

## remove molecules without gene annotation
NAind=which(res$gene_name_human_rna=="")
res=res[-NAind,]
dim(res) # 1010309    14
```

```
## stat
stat1=read.csv("../data/stat/1069_stats.csv",header=T,as.is=T)
keepInd=which(stat1$molecule_id!="")
stat1=stat1[keepInd,]
stat1=stat1[,!colnames(stat1)%in%"cluster_dada2"]
stat1$molecule_id=gsub("SAMPLE_1_MOLECULE","SAMPLE_1069_MOLECULE",stat1$molecule
_id)

stat2=read.csv("../data/shortRead3/1853/1853_sample_TP10-S1000T-
tumor_c_stats.csv",header=T,as.is=T)
keepInd=which(stat2$molecule_id!="")
stat2=stat2[keepInd,]

stat3=read.csv("../data/shortRead3/1853/1853_sample_TP10-S1000T-
tumor_d_stats.csv",header=T,as.is=T)
keepInd=which(stat3$molecule_id!="")
stat3=stat3[keepInd,]

stat4=read.csv("../data/shortRead3/1857/1857_sample_TP10-S1000T-
tumor_a_stats.csv",header=T,as.is=T)
keepInd=which(stat4$molecule_id!="")
stat4=stat4[keepInd,]

stat5=read.csv("../data/shortRead3/1857/1857_sample_TP10-S1000T-
tumor_b_stats.csv",header=T,as.is=T)
keepInd=which(stat5$molecule_id!="")
stat5=stat5[keepInd,]

stat=rbind(stat1,stat2,stat3,stat4,stat5)

sum(res$molecule_id%in%stat$molecule_id)  # 1010309

df=data.frame(molecule_id=stat$molecule_id, read_count=stat$read_count,
            gene=res[stat$molecule_id,"gene_name_human_rna"],
            isoform=res[stat$molecule_id,"ref_id_human_rna"],
            stringsAsFactors = F)
df[is.na(df$isoform),"isoform"]="NA"  # correct NA isoform
df=cbind(df,isoName=paste(df$gene,df$isoform,sep="__"))
dim(df)  # 1022111      5

## gene count
dfSplitGene=split(df,df$gene)
geneCount=sapply(dfSplitGene,function(x) return(sum(x[,2])))
length(geneCount)  # 8325
```

```
## isoform count
dfSplitIso=split(df,df$isoName)
isoCount=sapply(dfSplitIso,function(x) return(sum(x[,2])))
length(isoCount)  # 16784  including NA

gene=sapply(strsplit(names(isoCount),split="__"), function(x) return(x[1]))

tab=data.frame(Gene=gene,GeneCount=as.numeric(geneCount[gene]),
            Isoform=sapply(strsplit(names(isoCount),split="__"), function(x) return(x[2])),
            IsoformCount=as.numeric(isoCount))

write.csv(tab,file="count/sample_TP10-S1000T.count.csv",row.names=F,quote=F)


###################### TP10-S1000N, 3N

rm(list=ls())

res1=read.csv("../data/result3/1851_1179_mRNA_JianHua_AB105/1851_output/1851_sample
_S1000T-NORMAL_a/1851_sample_S1000T-NORMAL_a_stats_trimmed.csv",header=T,as.is=T)
dim(res1) # 176502     14

res2=read.csv("../data/result3/1851_1179_mRNA_JianHua_AB105/1851_output/1851_sample
_S1000T-NORMAL_b/1851_sample_S1000T-NORMAL_b_stats_trimmed.csv",header=T,as.is=T)
dim(res2) # 295923     14

res=rbind(res1,res2)
dim(res)  # 472425     14
rownames(res)=res$molecule_id

## remove molecules without gene annotation
NAind=which(res$gene_name_human_rna=="")
res=res[-NAind,]
dim(res) # 466674     14

## barcode stat file
stat1=read.csv("../data/shortRead3/1851/1851_sample_S1000T-
NORMAL_a_stats.csv",header=T,as.is=T)
keepInd=which(stat1$molecule_id!="")
stat1=stat1[keepInd,]

stat2=read.csv("../data/shortRead3/1851/1851_sample_S1000T-
NORMAL_b_stats.csv",header=T,as.is=T)
```

```
keepInd=which(stat2$molecule_id!="")
stat2=stat2[keepInd,]

stat=rbind(stat1,stat2)

sum(res$molecule_id%in%stat$molecule_id)  # 466674

df=data.frame(molecule_id=stat$molecule_id, read_count=stat$read_count,
            gene=res[stat$molecule_id,"gene_name_human_rna"],
            isoform=res[stat$molecule_id,"ref_id_human_rna"],
            stringsAsFactors = F)
df[is.na(df$isoform),"isoform"]="NA"  # correct NA isoform
df=cbind(df,isoName=paste(df$gene,df$isoform,sep="__"))
dim(df)  # 472425      5

## gene count
dfSplitGene=split(df,df$gene)
geneCount=sapply(dfSplitGene,function(x) return(sum(x[,2])))
length(geneCount)  # 6096

## isoform count
dfSplitIso=split(df,df$isoName)
isoCount=sapply(dfSplitIso,function(x) return(sum(x[,2])))
length(isoCount)  # 11370  including NA

gene=sapply(strsplit(names(isoCount),split="__"), function(x) return(x[1]))

tab=data.frame(Gene=gene,GeneCount=as.numeric(geneCount[gene]),
            Isoform=sapply(strsplit(names(isoCount),split="__"), function(x) return(x[2])),
            IsoformCount=as.numeric(isoCount))

write.csv(tab,file="count/sample_TP10-S1000N.count.csv",row.names=F,quote=F)
```

**Fusion text-searching**
See searchFusion.r script for details

########## searchFusion.r ##########

```
rm(list=ls())

getRevComp <- function(s){
  sSplit=strsplit(s,split="")[[1]]
```

```
  N=length(sSplit)
  sRevSplit=rep("",length=N)
  for(i in 1:N){
    if(sSplit[i]=="A"){
      sRevSplit[N-i+1]="T"
    }else if(sSplit[i]=="T"){
      sRevSplit[N-i+1]="A"
    }else if(sSplit[i]=="C"){
      sRevSplit[N-i+1]="G"
    }else if(sSplit[i]=="G"){
      sRevSplit[N-i+1]="C"
    }else{
      sRevSplit[N-i+1]="N"
    }
  }
  sRev=paste(sRevSplit,collapse="")
  return(sRev)
}

#FileList=list.files("/zfs1/sliu/Luo/LoopSeq/dataAll")


res=read.csv("Unique_fusion_V3.csv",header=T,as.is=T)

for(i in 1:nrow(res)){
  print(i)

  seqSplit=strsplit(res[i,"fusionSeq"],split="")[[1]]

  ### forward seq
  seqF=toupper(paste(seqSplit[c(41:50,52:61)],collapse=""))
  ### reverse seq
  seqR=getRevComp(seqF)

  ## search among the data
  #BAMfile=paste("/zfs1/sliu/Luo/LoopSeq/dataAll/*/sample_T*/contig_list_trimmed.fa",sep="")
  BAMfile=paste("/zfs2/sliu/Luo/LoopSeq/pipeline3/data/*/TP*fastq",sep="")

  s=paste("grep -n ",seqF," ",BAMfile," | awk -F \":\" '{print  \"", res[i,"Gene_A"], "\" \"\\t\" \"", 
res[i,"Gene_B"], "\" \"\\t\" $1 \"\\t\" $2 \"\\t\" $3 }' >> forward.txt",sep="")
  system(s)

  s=paste("grep -n ",seqR," ",BAMfile," | awk -F \":\" '{print  \"", res[i,"Gene_A"], "\" \"\\t\" \"", 
res[i,"Gene_B"], "\" \"\\t\" $1 \"\\t\" $2 \"\\t\" $3 }' >> reverse.txt",sep="")
```

```
  system(s)

}
```

## Supplemental figure S1

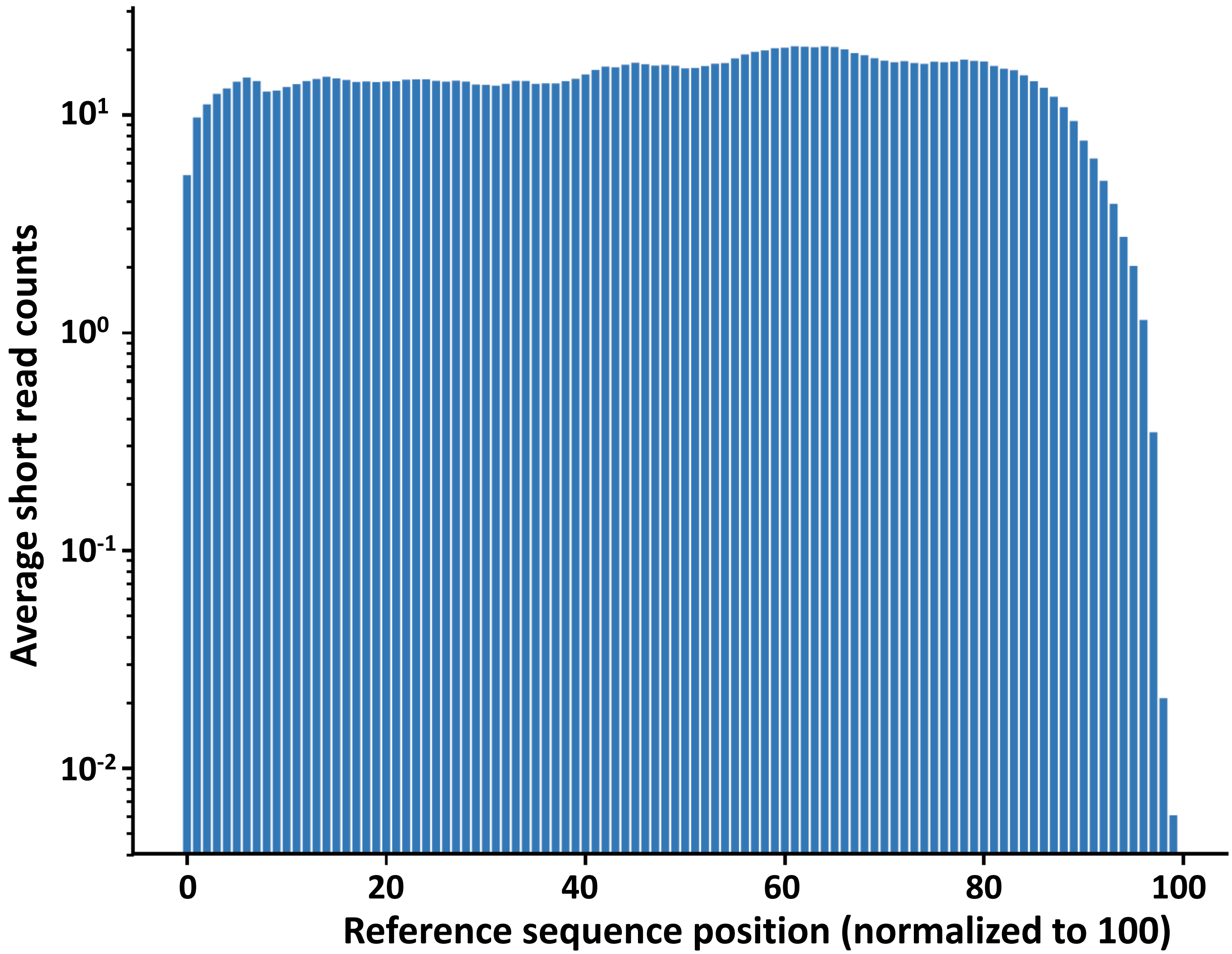

**Supplemental figure S2**

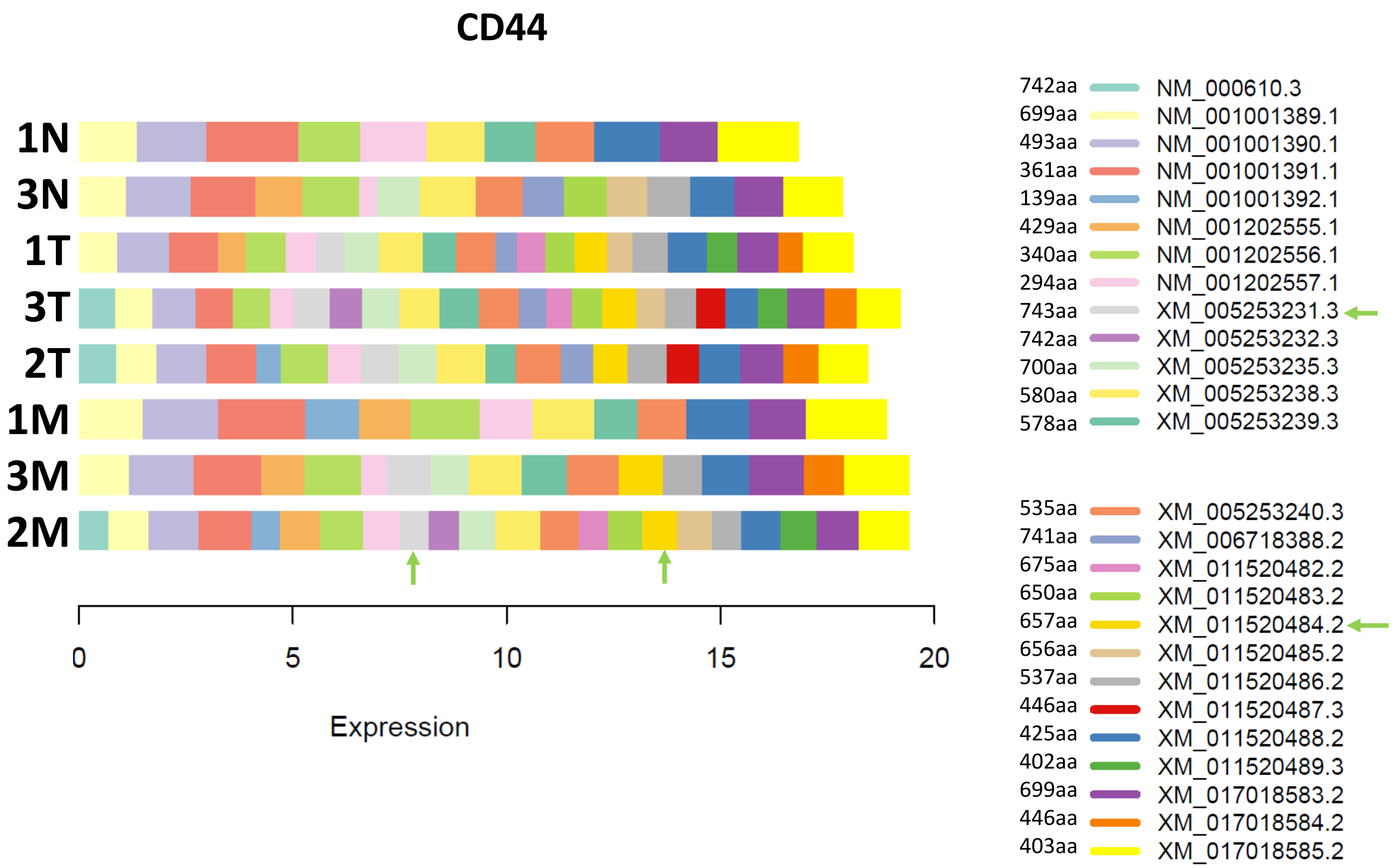

## Supplemental figure S3

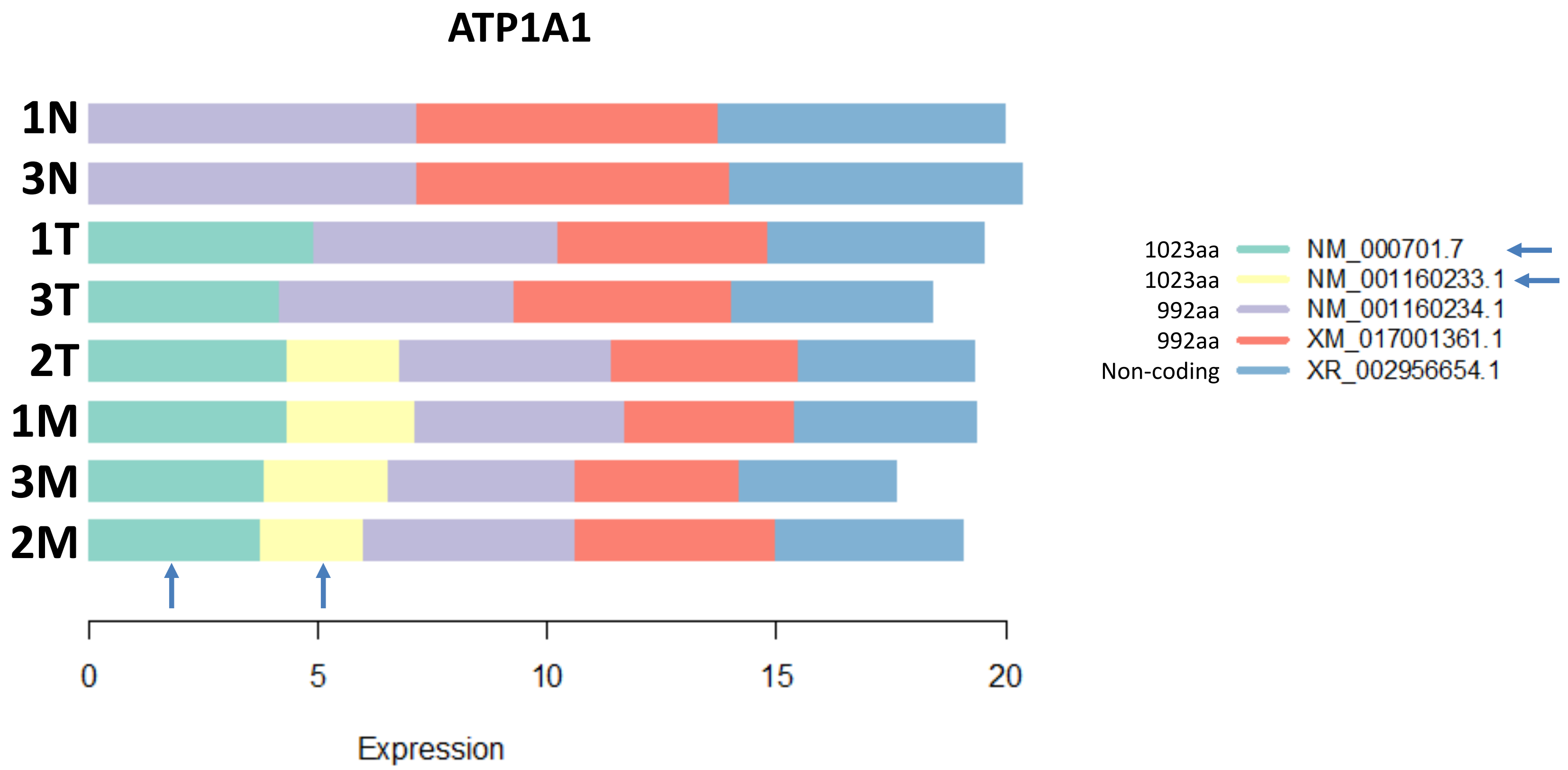

# Supplemental figure S4

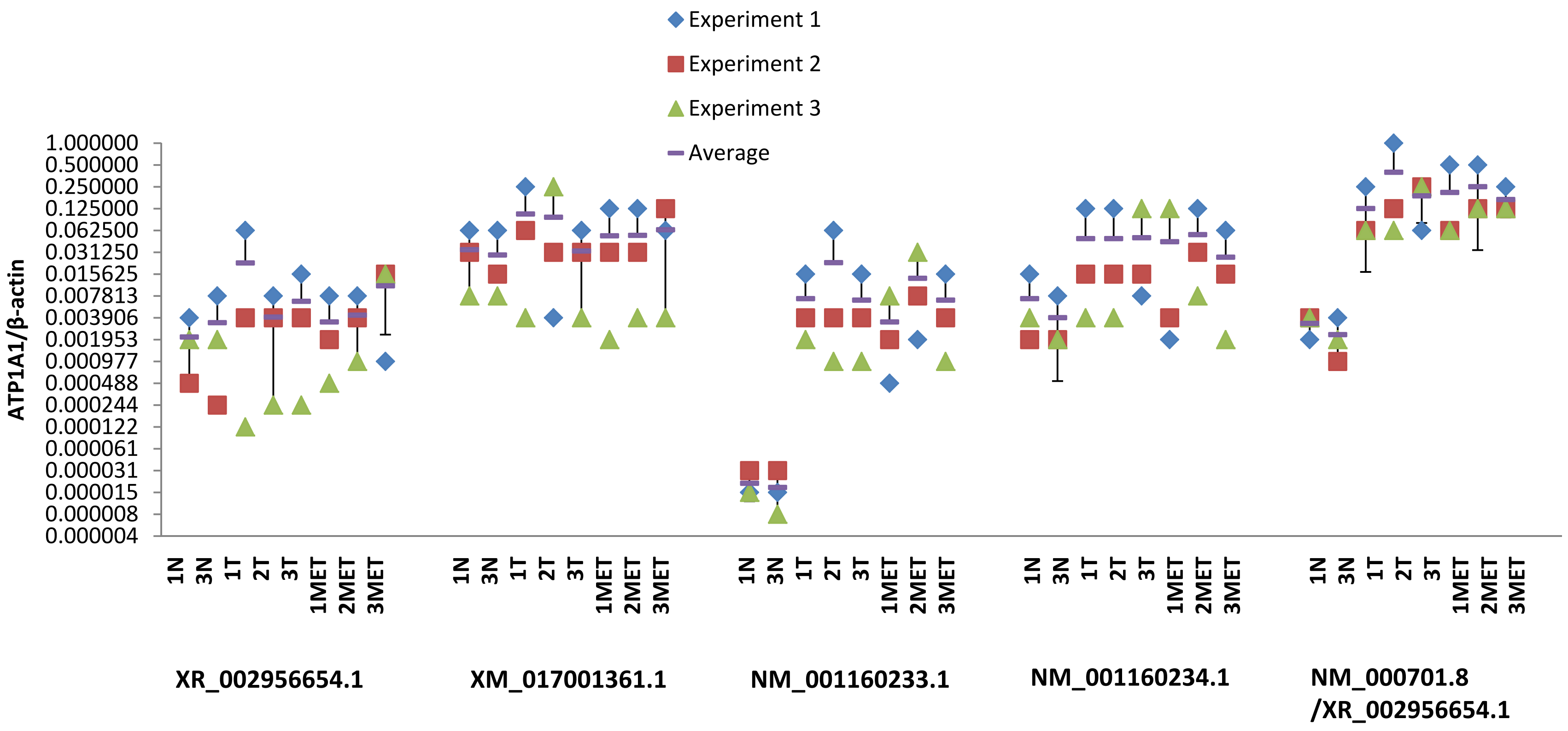

## Supplemental figure S5

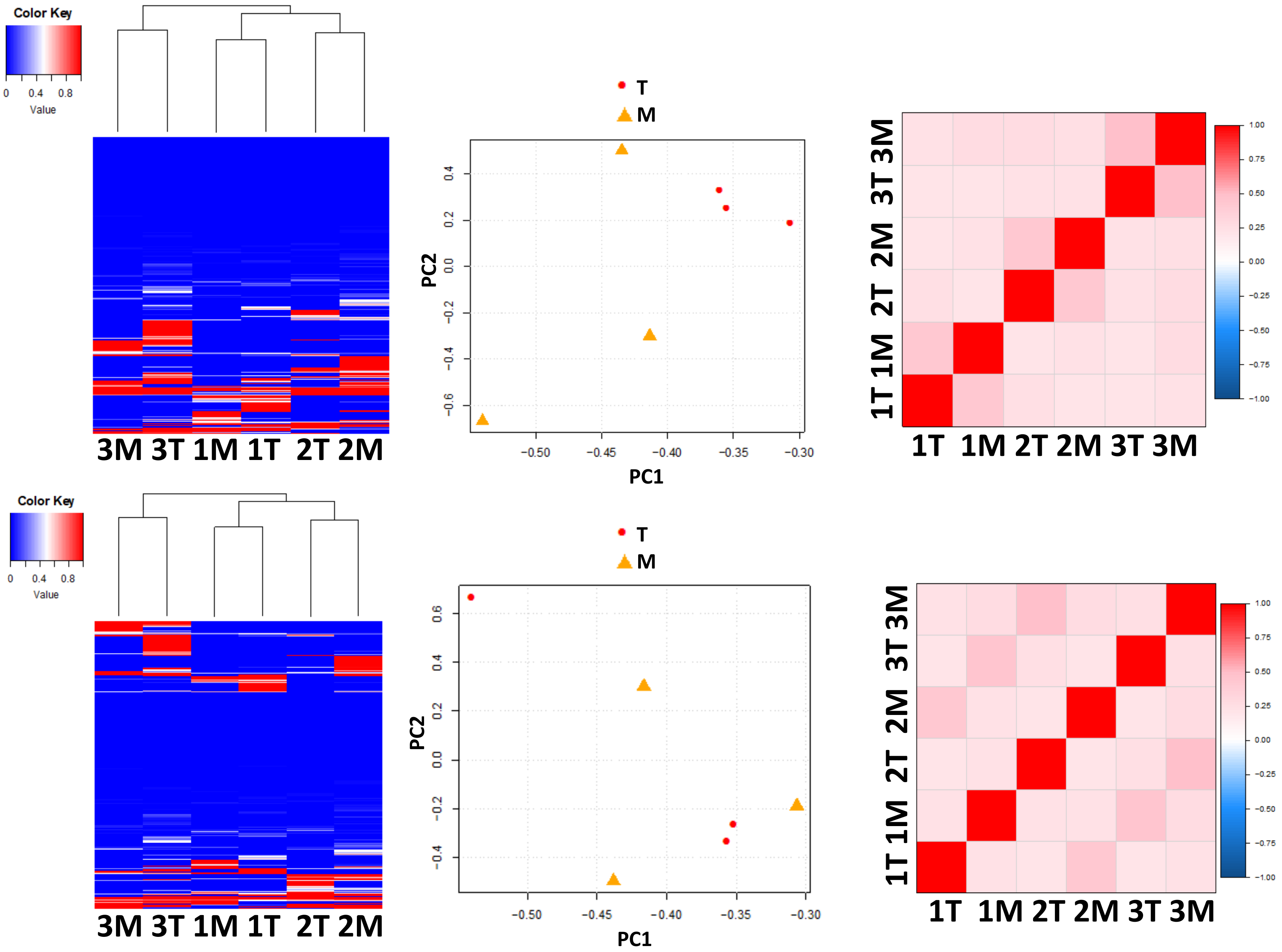

**Supplemental figure S6**

# Detection of fusion transcripts

## Criteria for filtering chromosome rearrangement based fusion transcripts after SQANTI mapping

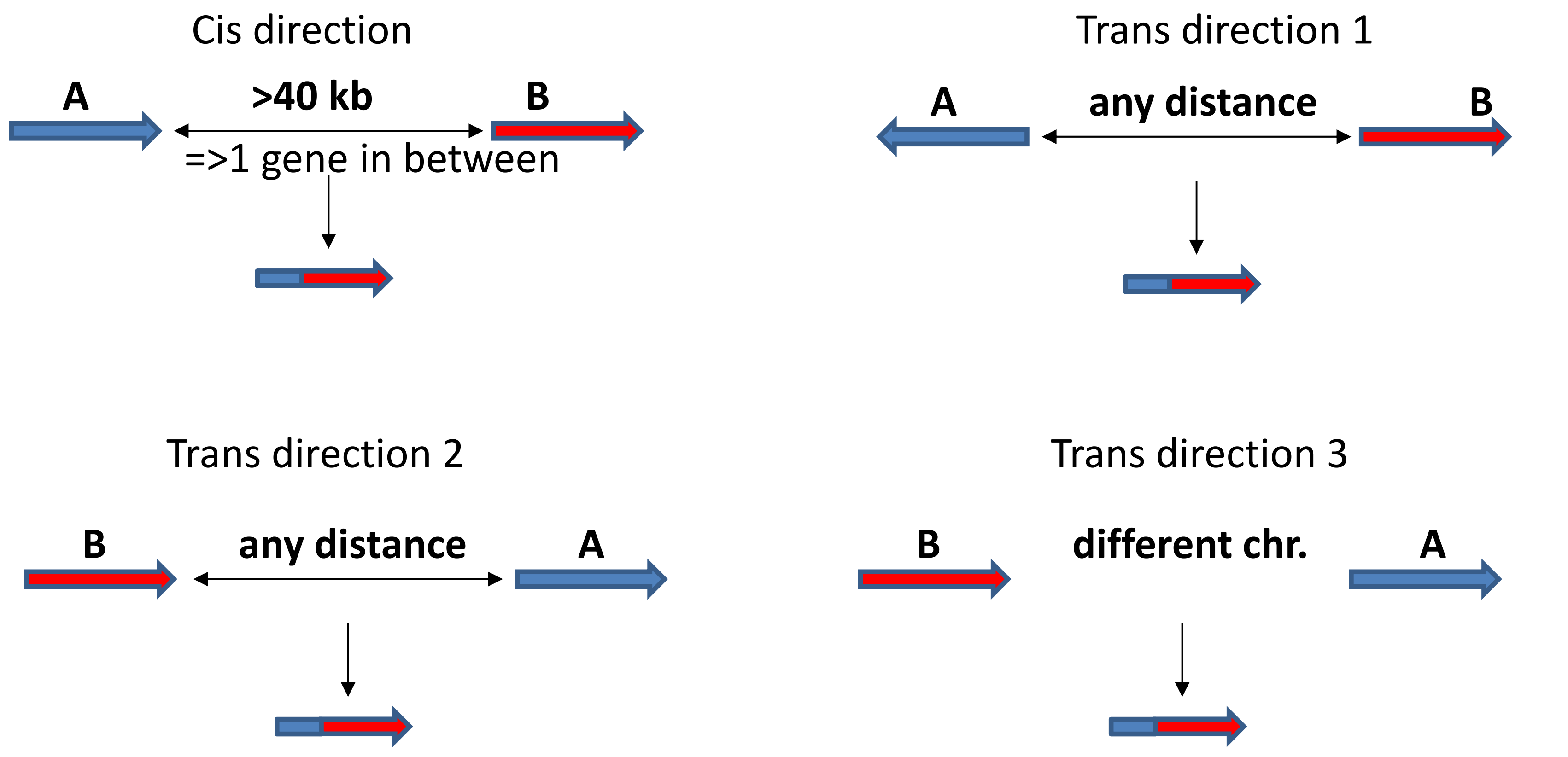

## Supplemental figure S7

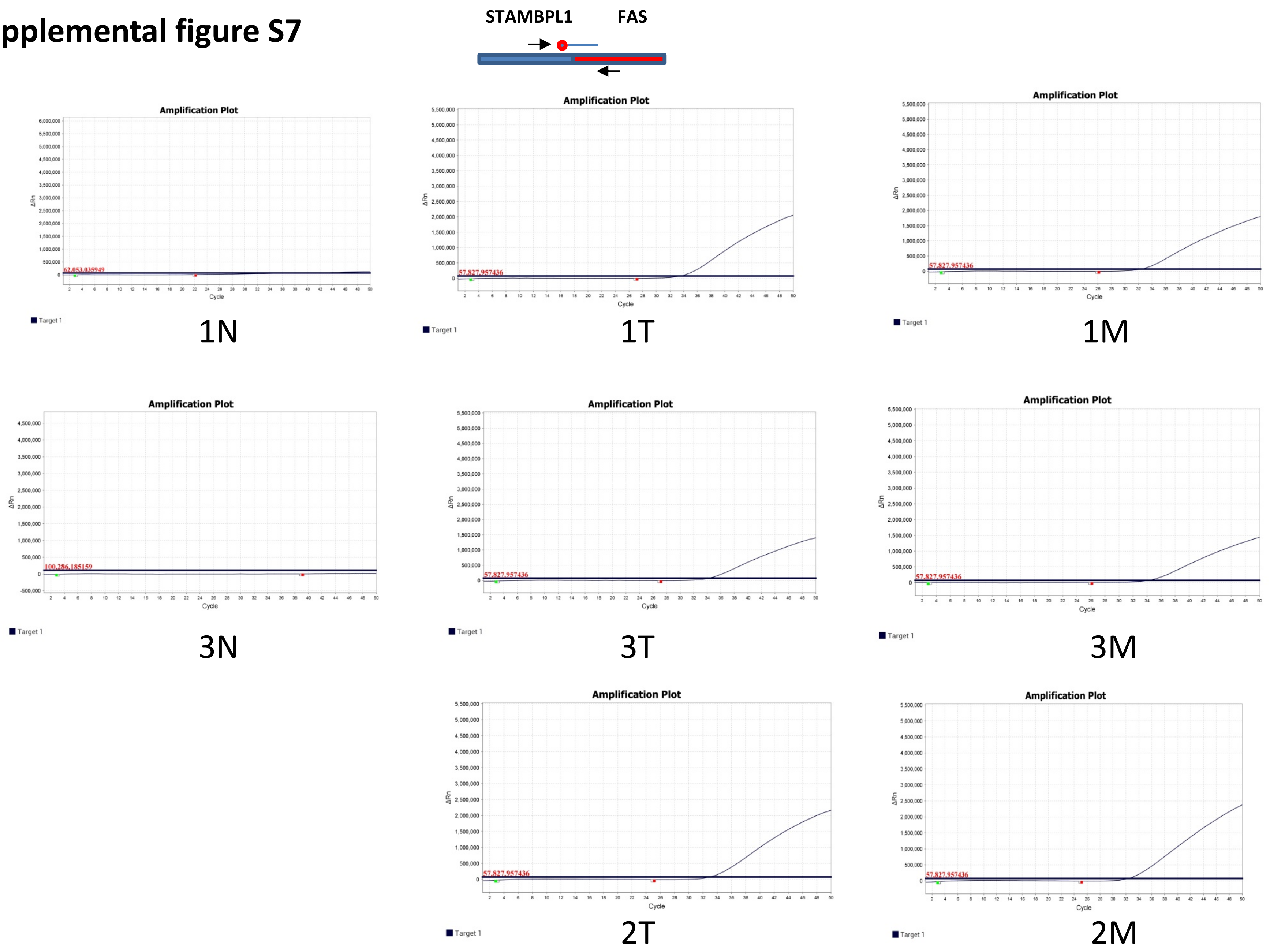

## Supplemental figure S8

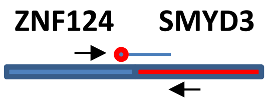


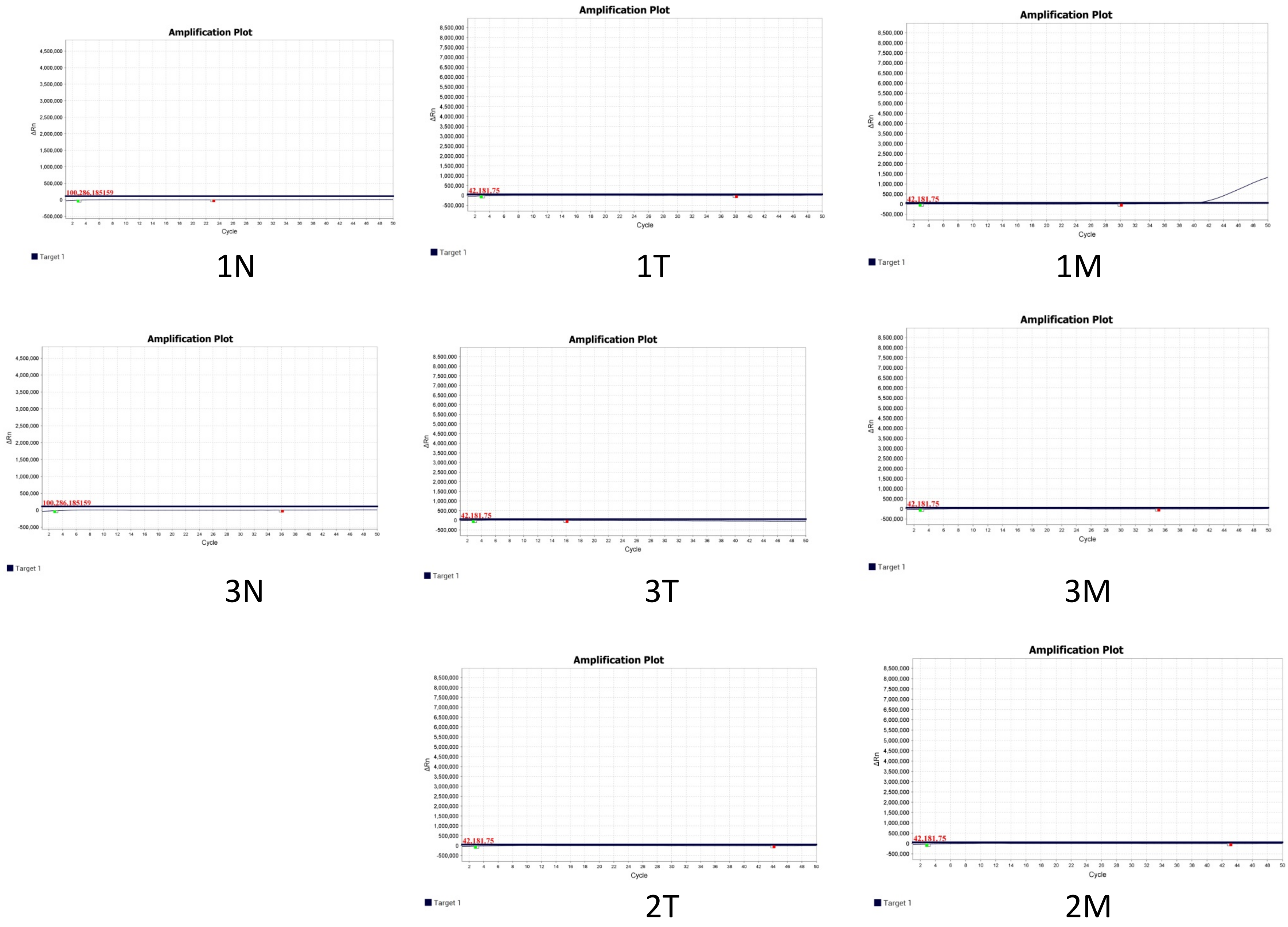

# Supplemental figure S9

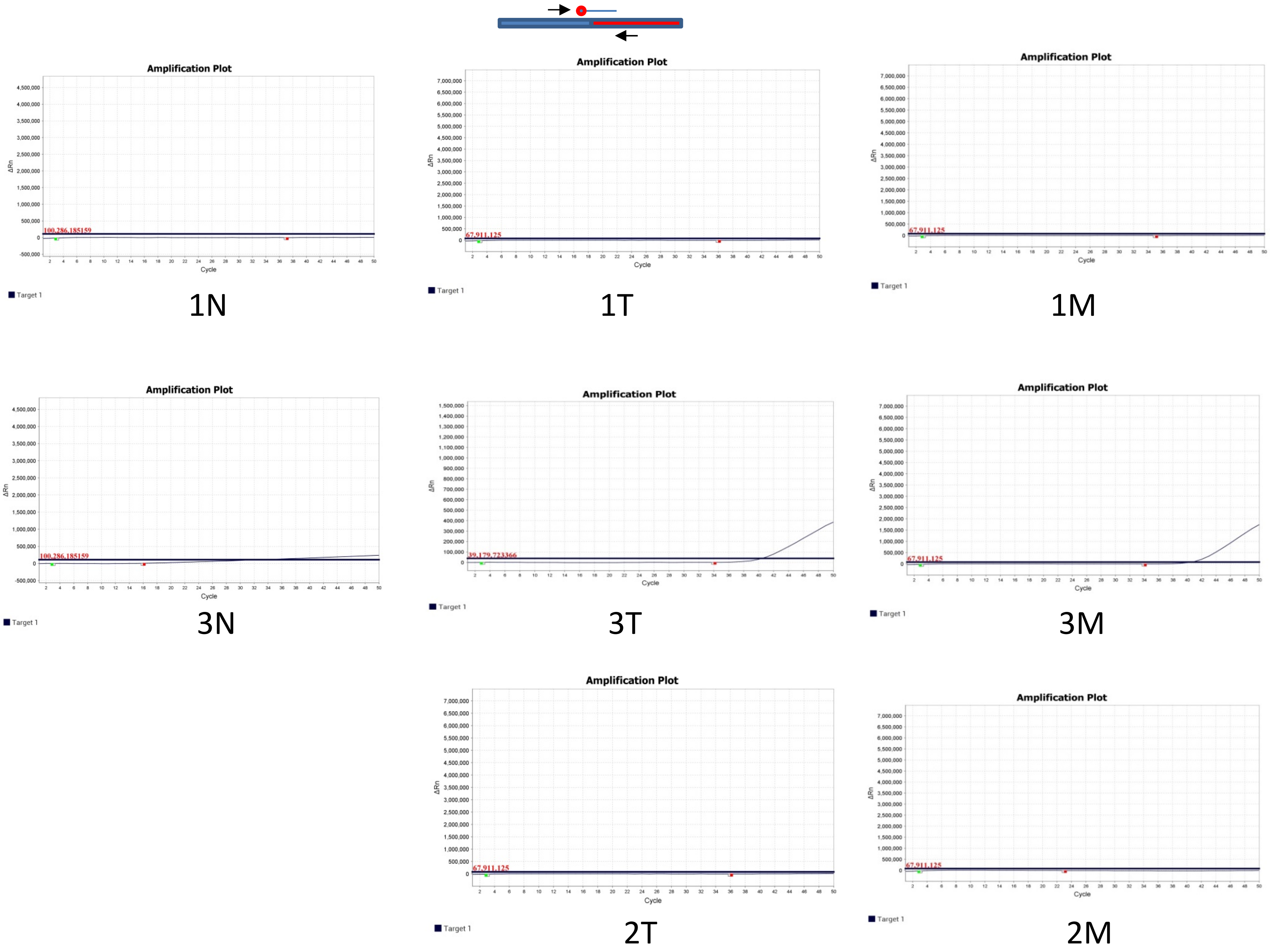

# Supplemental figure S10

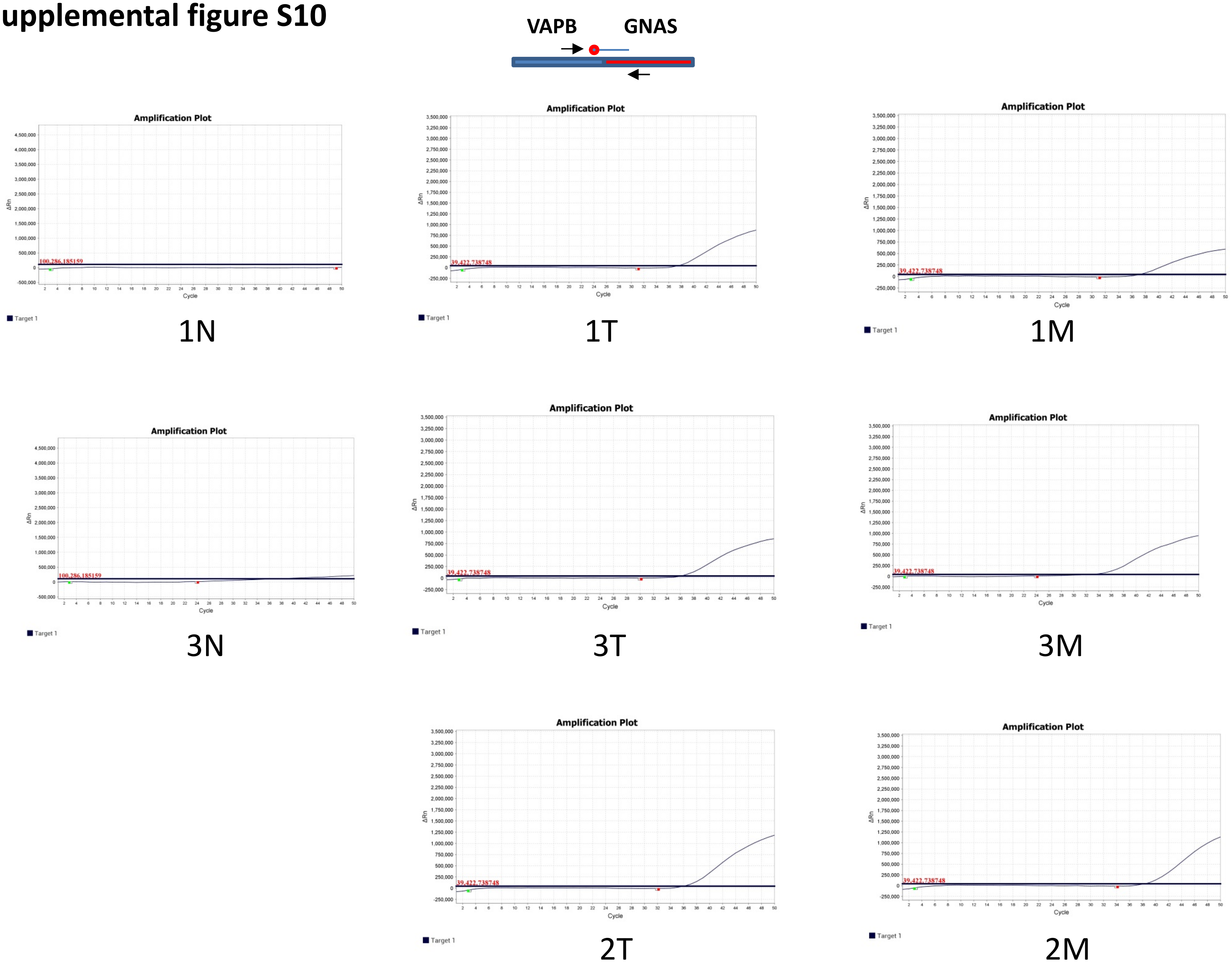

# Supplemental figure S11

## β-ACTIN

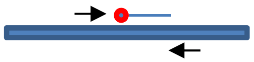

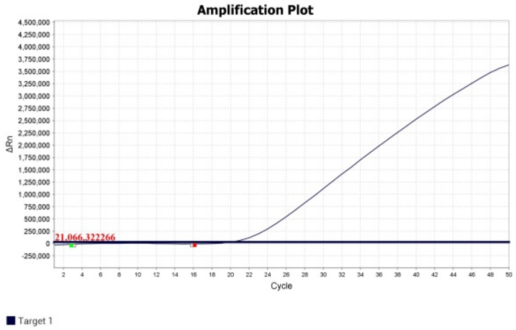


1N

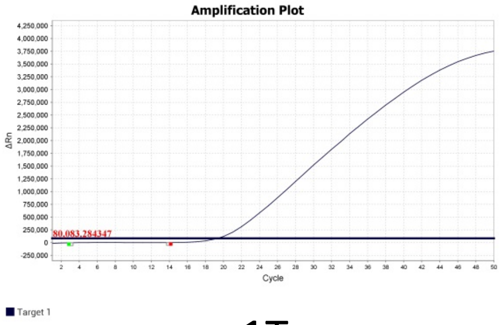


1T

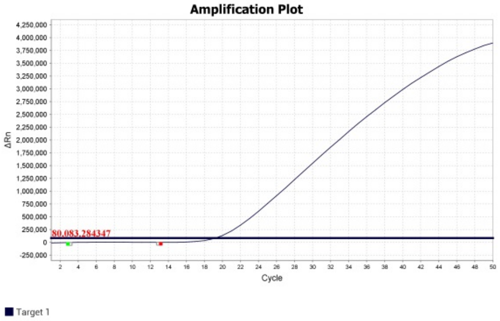


1M

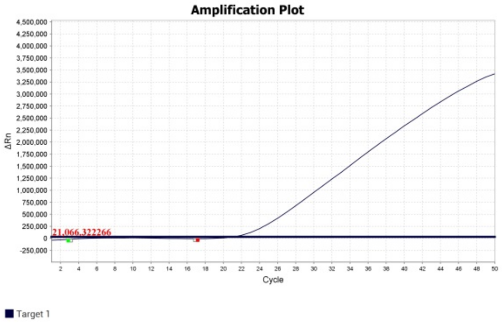


3N

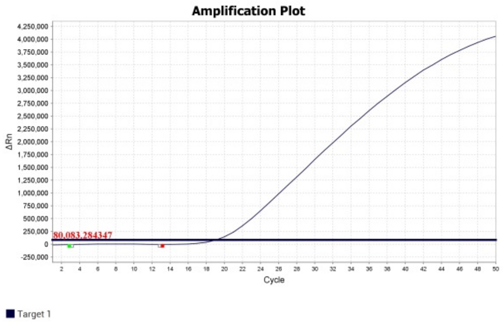


3T

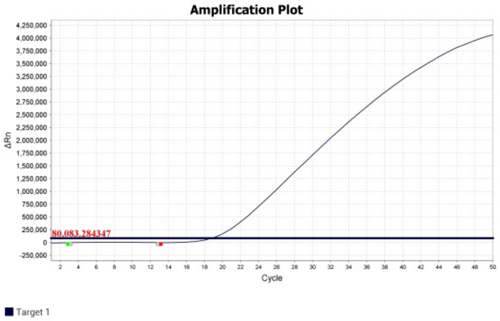


3M

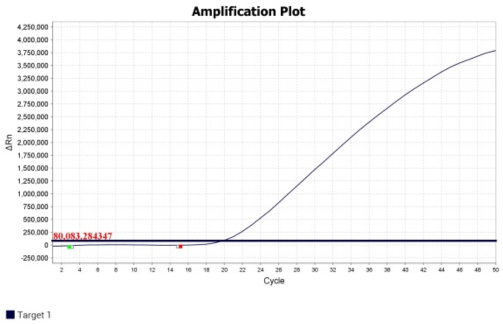


2T

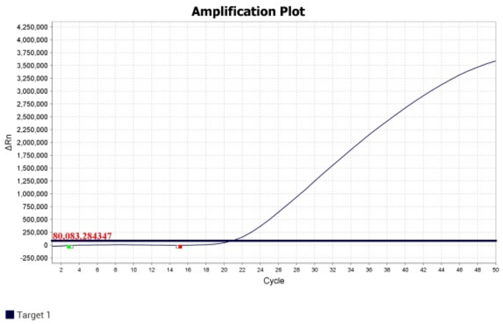


2M
