# Supplementary material for: Targeted transcriptome analysis using synthetic long read sequencing uncovers isoform reprograming in the progression of colon cancer
Source: Commun Biol. 2021 Apr 27;4:506. doi: 10.1038/s42003-021-02024-1 (PMC8079361; doi:10.1038/s42003-021-02024-1)
Supplement: Supplementary file 3 — Description of Additional Supplementary Files. [file 42003_2021_2024_MOESM3_ESM.pdf]

Description of Additional Supplementary Files

File Name: Supplementary Data 1

Description: Sequencing error of LoopSeq and Illumina on ERCC sample. S1A: Sequencing error per site. S1B: Pearson correlation comparing LoopSeq and Illumina sequencing error.

File Name: Supplementary Data 2

Description: Target sequence selection.

File Name: Supplementary Data 3

Description: Gene short-read count.

File Name: Supplementary Data 4

Description: Differentially expressed genes.

File Name: Supplementary Data 5

Description: Differentially expressed isoforms.

File Name: Supplementary Data 6

Description: Differentially expressed genes and isoforms.

File Name: Supplementary Data 7

Description: Total SNVs identified in colon cancer samples.

File Name: Supplementary Data 8

Description: Unevenly distributed SNV isoforms within a gene.

File Name: Supplementary Data 9

Description: Differential expression of SNV isoforms compared with wild type counterparts.

File Name: Supplementary Data 10

Description: SNV isoform validation via targeted RNA sequencing. S10A: Gene FAM104A. S10B: Gene PABPC1.

File Name: Supplementary Data 11

Description: Switching SNV isoforms between tumor and metastasis.

File Name: Supplementary Data 12

Description: Colon cancer related mutations/SNVs.

File Name: Supplementary Data 13

Description: Supporting reads for novel fusion detection. S13A: Summary table. S13B: STAMBPL1 - FAS (1M). S13C: STAMBPL1 - FAS (2M). S13D: SMYD3 – ZNF124 (3L). S13E: ECHDC1 – PTPRK (3T). S13F: ECHDC1 – PTPRK (3L).

File Name: Supplementary Data 14

Description: LoopSeq targeted sequencing on-target rate.

File Name: Supplementary Data 15

Description: Primers and probes design.
