# Supplementary material for: Targeted transcriptome analysis using synthetic long read sequencing uncovers isoform reprograming in the progression of colon cancer
Source: Commun Biol. 2021 Apr 27;4:506. doi: 10.1038/s42003-021-02024-1 (PMC8079361; doi:10.1038/s42003-021-02024-1)
Supplement: Supplementary file 18 — Supplementary Data 15 [file 42003_2021_2024_MOESM18_ESM.docx]

**Supplementary Data 15**

**Primers and probes design**

Gene Isoform Primer Probe

**ATP1A1** **XR_002956654.1 CTCCGGTAACGGTGACGTTTC 5’-FAM/CCATGGTGG/ZEN/CGGTGCTCAGT/3IABkFQ**

/CGCCTCCTCCTCACTTCCTAA

**XM_017001361.1 GTCCTCTCATAACGCATATGCTG 5’-FAM/TGTCTCTAA/ZEN/CCAGAGCAATCTGG/3IABkFQ**

/GCTGACCACCTTACCCTCCT

**NM_001160233.1 GCCCTCCCCCAAAGAAAAAAC 5’-FAM/TTCTAAGTG/ZEN/CGAAGCCGGCTG/3IABkFQ**

**/ACACACCCTTTCTGGCACCAG**

**NM_001160234.1 TAAAAACATGAAATGAGGTTGGCAC 5’-FAM/CTTTATCTC/ZEN/CACGCTGTGGAAG/3IABkFQ**

**/CCAGCAGTGTATCCTTAGGCAAG**

**NM_000701.8/ GAGCTGCTCTGTGCTTTTCT 5’-FAM/TGATTCTCC/ZEN/AGCGACAGGACC/3IABkF**

**XR_002956654.1 /CCATGGTGGCGGTGCTCAGT**

**STAMBPL1 XM_011539985.2 TTCATCCACACACCAAGGAGC 5’-FAM/CAGGCTGTT/ZEN/CAGTATGCTCAG/3IABkFQ**

**/FAS /XM_011539766.2 /TGTGCCAGCCTTGTGCACACA**

**ZNF124 XM_005273257.4 GATGTCGGGACACCCCGGAA 5’-FAM/CTGGGAAAT/ZEN/GGAAGGAAAAGCT/3IABkFQ**

**/SMYD3 /NM_001375963.1 /GGCACTGAGAGCATCGCATC**

**PTPRK XM_011536021.3 GGATCGGCCCAAGGCCAGT 5’-FAM/CTCCGCAGG/ZEN/TTAATGACTCCA/3IABkFQ**

**/ECHDC1 /NM_001105544.1 /GTGGACGAATCTGATCTTACTC**

**VAPB NM_001195677.2 GGATCGGCCCAAGGCCAGT 5’-FAM/CTCCGCAGG/ZEN/TTAATGACTCCA/3IABkFQ**

**/GNAS /XM_017027814.2 /GTGGACGAATCTGATCTTACTC**
